# Receptive prosody in nonfluent primary progressive aphasias

**DOI:** 10.1016/j.cortex.2010.09.004

**Published:** 2012-03

**Authors:** Jonathan D. Rohrer, Disa Sauter, Sophie Scott, Martin N. Rossor, Jason D. Warren

**Affiliations:** a Dementia Research Centre, Department of Neurodegenerative Disease, UCL Institute of Neurology, University College London, UK; b Institute of Cognitive Neuroscience, UCL Institute of Neurology, University College London, UK

**Keywords:** Primary progressive aphasia, Frontotemporal dementia, Frontotemporal lobar degeneration, Logopenic aphasia, Progranulin, Prosody

## Abstract

**Introduction:**

Prosody has been little studied in the primary progressive aphasias (PPAs), a group of neurodegenerative disorders presenting with progressive language impairment.

**Methods:**

Here we conducted a systematic investigation of different dimensions of prosody processing (acoustic, linguistic and emotional) in a cohort of 19 patients with nonfluent PPA syndromes (11 with progressive nonfluent aphasia, PNFA; five with progressive logopenic/phonological aphasia, LPA; three with progranulin-associated aphasia, GRN-PPA) compared with a group of healthy older controls. Voxel-based morphometry (VBM) was used to identify neuroanatomical associations of prosodic functions.

**Results:**

Broadly comparable receptive prosodic deficits were exhibited by the PNFA, LPA and GRN-PPA subgroups, for acoustic, linguistic and affective dimensions of prosodic analysis. Discrimination of prosodic contours was significantly more impaired than discrimination of simple acoustic cues, and discrimination of intonation was significantly more impaired than discrimination of stress at phrasal level. Recognition of vocal emotions was more impaired than recognition of facial expressions for the PPA cohort, and recognition of certain emotions (in particular, disgust and fear) was relatively more impaired than others (sadness, surprise). VBM revealed atrophy associated with acoustic and linguistic prosody impairments in a distributed cortical network including areas likely to be involved in perceptual analysis of vocalisations (posterior temporal and inferior parietal cortices) and working memory (fronto-parietal circuitry). Grey matter associations of emotional prosody processing were identified for negative emotions (disgust, fear, sadness) in a broadly overlapping network of frontal, temporal, limbic and parietal areas.

**Conclusions:**

Taken together, the findings show that receptive prosody is impaired in nonfluent PPA syndromes, and suggest a generic early perceptual deficit of prosodic signal analysis with additional relatively specific deficits (recognition of particular vocal emotions).

## 1 Introduction

Primary progressive aphasia (PPA) is a group of neurodegenerative disorders which presents with impairment of language (Mesulam, 2001, 2003). Several canonical subtypes have been identified: semantic dementia (SD), led by verbal semantic impairment; progressive nonfluent aphasia (PNFA), led by, apraxia of speech and agrammatism; progressive logopenic/phonological aphasia (LPA) led by word-finding difficulty with impaired sentence repetition and comprehension (Gorno-Tempini et al., 2004, 2008); and an aphasic syndrome associated with mutations in the progranulin (*GRN*) gene (progranulin-associated aphasia, GRN-PPA), which shares some features of LPA but with expressive agrammatism and more marked semantic impairment (Rohrer et al., 2010a, 2010b). Whereas the production and processing of verbal material in PPA have been extensively studied, less attention has been paid to nonverbal aspects of vocal communication. Expressive prosody, or the ‘melody’ of speech, is abnormal in many patients with PPA (Josephs et al., 2006): apraxia of speech or expressive agrammatism in PNFA, and word-finding pauses in LPA tend to disrupt the rhythm and intonational structure of utterances, rendering their speech dysprosodic. However, it is not clear whether such patients have an underlying deficit in the comprehension of prosody, ‘receptive dysprosodia’ (Ross, 1981). This issue is of both neurobiological and clinical importance: neurobiologically, such a deficit would signify a pervasive derangement in the processing of vocal signals in PPA, while clinically, there would be important implications for everyday communication. Prosody is complex and conveys multidimensional information about the speaker’s intentions and emotional state, while facilitating disambiguation of the meaning of an utterance (e.g., statement *vs* question). At the most fundamental acoustic level, prosody comprehension depends on an ability to process variations in vocal pitch, duration and intensity (loudness) that constitute the building blocks of prosodic contours. Processing of prosodic patterns in words, phrases and sentences is required to determine lexical stress and declarative versus interrogative intention (linguistic prosody). Representation of vocal affective information is required to decode the speaker’s emotional state (emotional prosody).

Here we conducted a systematic investigation of different dimensions of prosody processing (acoustic, linguistic and emotional) in a cohort of patients with PPA versus healthy older control subjects. For the purposes of this study, we focus on nonfluent variants of PPA rather than SD. ‘Nonfluent’ is a problematic term but is used here as elsewhere in the PPA literature, i.e., to indicate reduced overall quantity of speech produced. Patients with nonfluent PPA (unlike patients with SD) show deficits in the perceptual analysis of nonverbal environmental sounds (Goll et al., 2010): the nonfluent PPA variants are therefore the logical initial target for an investigation of prosody processing. Here we used voxel-based morphometry (VBM) to identify neuroanatomical associations of prosodic functions in the nonfluent PPA syndromes.

## 2 Methods

Nineteen consecutive patients with a diagnosis of nonfluent PPA (11 with PNFA, five with LPA, three with GAA) were recruited. All patients fulfilled a diagnosis of PPA based on a clinical presentation led by progressive language impairment without generalised intellectual decline, and diagnosis for each subgroup was based on the following neuropsychological criteria (described in detail in Rohrer et al., 2010b): for PNFA, reduced speech rate with apraxia of speech, speech production errors and agrammatism, and relatively preserved single word comprehension; for LPA, anomia with prolonged word-finding pauses (but relatively spared single word repetition and comprehension) and impaired sentence repetition and comprehension, without speech apraxia or expressive agrammatism; for GRN-PPA, anomia with impaired single word comprehension, impaired sentence comprehension and repetition, and expressive agrammatism without speech apraxia, associated with a mutation in the *GRN* gene. These criteria are in line with criteria for PPA previously proposed by other authors (Neary et al., 1998; McKhann et al., 2001; Gorno-Tempini et al., 2004, 2008). Fourteen cognitively normal control subjects also participated in the study. One patient (with LPA) had known mild industrial hearing loss; peripheral hearing was assessed in relation to age norms using pure tone audiometry in 17 patients, and subclinical peripheral hearing loss involving speech frequencies (below 4000 Hz) was detected in a further two cases (both with PNFA). All patients had an initial general neuropsychological assessment including tests of single word comprehension (the Warrington Synonyms test, Warrington et al., 1998), executive function (Trail Making Test, Reitan, 1959) and digit span: differential performance in these domains might in principle drive differences between PPA subgroups on tests of receptive prosody requiring auditory short-term memory or matching to verbal alternatives (see below). Demographic and neuropsychological data are summarised in Table 1: the PPA group performed significantly worse than controls on all tests, while the only significant difference between the disease subgroups was more impaired single word comprehension in LPA compared with PNFA and lower forwards digit span in GRN-PPA compared to the other subgroups. All patients except one with GRN-PPA who had a cardiac pacemaker underwent magnetic resonance (MR) brain imaging on a 1.5 T GE Signa scanner (General Electric, Milwaukee, WI). T1-weighted volumetric images were obtained with a 24-cm field of view and 256 × 256 matrix to provide 124 contiguous 1.5-mm-thick slices in the coronal plane. Magnetic resonance imaging (MRI) brain scans were also acquired for the healthy control group using the same acquisition protocol, providing a normal comparison group for assessment of PPA-related atrophy in the VBM analysis (see below). Research ethics approval for this study was obtained from the National Hospital for Neurology and Neurosurgery and University College London Hospitals Research Ethics Committees.

All subjects were assessed using a battery of experimental tests probing different aspects of receptive prosody. All stimuli were prepared or recorded as digital wavefiles from a notebook computer via AKG K141 Monitor^®^ headphones, at comfortable listening level in a quiet room. Several practice trials were given for each test, to ensure subjects understood the task; no feedback was given about performance during the test. For all experiments, stimulus order was randomised with respect to the prosody parameter of interest.

### 2.1 Experiment 1: Acoustic processing of prosody components

**Fig. 1:**
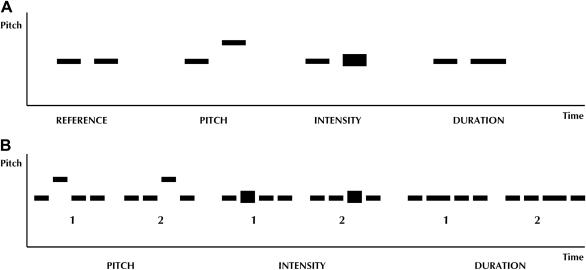
Diagram showing the design of Experiment 1, assessing acoustic processing of prosodic components: (A) pair discrimination – subjects heard either a pair of syllables of same pitch, duration and intensity or two pairs of differing pitch, intensity (represented by thicker rectangle) or duration; and (B) contour discrimination – subjects heard two 4-syllable sequences (1 and 2, in randomised order) for either pitch, intensity or duration and were asked to say whether same or different. Stimulus parameters were as follows: pitch values 120 or 160 Hz; intensity shifts 65% of average root-mean-square intensity for the syllable sequence; syllable duration values 500 or 1000 msec, inter-syllable duration 200 msec (see text for further details).

The structure of the experimental tasks is schematised in Fig. 1.

#### 2.1.1 Pair discrimination task (match/non-match, 12 trials)

Subjects were presented with pairs of CV syllables (‘ba’). On half the trials, syllables contained a single difference in pitch, intensity or duration; on the remaining trials the syllables were acoustically identical. Stimulus parameters were digitally manipulated using Matlab7.0^©^ (www.mathworks.com); pitch was manipulated using a previously described algorithm (von Kriegstein et al., 2006). The prosody variations used were intended to be easily detectable by normal subjects (see Fig. 1 for stimulus parameters). The task on each trial was to decide whether the two sounds were the same or different (i.e., a ‘match’ *vs* ‘non-match’ design).

#### 2.1.2 Contour discrimination task (match/non-match, 12 trials)

Subjects were presented with pairs of short (4-item) sequences using the same CV syllables as in the pair discrimination task, where each sequence in the pair contained a change in pitch, intensity or duration (parameters as in the pair discrimination task), but this change occurred at either of two positions (position 2 or 3) with equal probability. The task was to decide whether the two prosodic (pitch, intensity or duration) contours in each pair were the same or different.

### 2.2 Experiment 2: Linguistic prosody

Linguistic prosody test stimuli were adapted from Peppé and McCann (2003).

#### 2.2.1 Stress discrimination task (2 alternative forced choice, 14 trials)

Subjects heard a spoken phrase of the type: ‘**black** and blue’ [stressed word in bold] and were asked to decide whether the first or second colour term in the phrase was stressed.

#### 2.2.2 Intonation discrimination task (2 alternative forced choice, 14 trials)

Subjects heard a two-syllable word (name of a food) spoken either declaratively or interrogatively (e.g., ‘apple’ *vs* ‘apple?’). The subject’s task was to decide whether what they heard was a statement (as if read from a list) or a question (as if they were being asked if they wanted the food).

### 2.3 Experiment 3: Emotional (affective) prosody (6 alternative forced choice, 24 trials)

This experiment was adapted from Sauter (2006), based on a previously normed set of vocal emotional stimuli. Subjects heard a semantically neutral three-digit number (e.g., ‘one hundred and forty-seven’) recorded by an actor and spoken to convey one of six basic emotions (happiness, surprise, fear, sadness, disgust, anger; the set of sounds representing ‘happiness’ were spoken to convey either amusement or achievement). For each of the six emotions, four trials representing that emotion were administered; stimuli that were most consistently identified as representing that vocal emotion by the previous group of healthy control subjects (Sauter, 2006) were selected. The task on each trial was to decide which of the six basic emotions was represented in the vocalisation.

The modality specificity of any affective prosodic deficit was investigated using the same task for a parallel set of 24 facial expression stimuli [four trials representing each of the same six canonical emotions, derived from the set created by Ekman and Friesen (1976), which has been widely assessed in both healthy and clinical populations]. These facial expression stimuli were administered to 13 of the 19 patients (as part of a separate study) in the timeframe of the prosody assessment; these patients represented each of the PPA subgroups (six PNFA, five LPA, two GRN-PPA). Facial emotion recognition in patients was assessed in relation to a group of 15 healthy age-matched control subjects.

#### 2.3.1 Behavioural analysis

Behavioural data were analysed statistically using STATA 10.0 (Stata Corporation, College Station, TX). Linear regression models were used to compare performance on the tests between groups after adjusting for age. 95% bias-corrected bootstrap confidence intervals with 1000 replicates were used (these methods make fewer assumptions about the underlying structure of the data than conventional analytical parametric tests). To look at within disease group comparisons Wilcoxon signed-rank tests were used to assess differences between patient scores as a percentage of the control mean.

#### 2.3.2 VBM analysis

To investigate the neuroanatomical associations of receptive prosody in the PPA group, a VBM analysis was performed using SPM5 software (http://www.fil.ion.ucl.ac.uk/spm) with default settings for all parameters. The patients’ MR brain images underwent an initial segmentation process in SPM5 which simultaneously estimated transformation parameters for warping grey matter (GM), white matter (WM) and cerebrospinal fluid (CSF) tissue probability maps (TPMs) onto the images. The native space GM segments were then rigidly spatially normalised, using just the rotations and translations from the inverse of the TPM transformation, and resampled to 1.5 mm isotropic resolution. These “imported” images were then iteratively warped to an evolving estimate of their group-wise GM average template using the DARTEL toolbox (Ashburner, 2007; Ashburner and Friston, 2009). The GM segmentations were then normalised using the final DARTEL transformations and modulated to account for volume changes. Finally, the images were smoothed using a 6 mm full-width at half-maximum (FWHM) Gaussian kernel. Linear regression models were used to examine changes in GM volume as functions of acoustic processing subtest scores, linguistic subtest scores and individual emotion scores. Voxel intensity was modelled as a function of score with subject age and total intracranial volume included as nuisance covariates. In order to reduce the likelihood of observing spurious prosody performance associations, whole brain analyses were masked inclusively by the region of PPA-associated atrophy, i.e., all those brain voxels showing significantly greater GM intensity in healthy controls than in the PPA group (thresholded at *p* < .01 uncorrected). Statistical parametric maps were displayed as overlays on a study-specific template, created by warping all native space whole-brain images to the final DARTEL template and calculating the average of the warped brain images.

## 3 Results

### 3.1 Behavioural data

On all acoustic processing and linguistic prosody subtests, the LPA subgroup performed significantly worse than controls (Table 2). The PNFA and *GRN*-PPA subgroups were significantly worse than controls on all subtests apart from stress discrimination (Table 2). The LPA group performed significantly worse than the PNFA group on the pair and intonation discrimination subtests, and worse than the *GRN*-PPA group on the pair and stress discrimination subtests. For the PPA group as a whole, performance was significantly worse on contour discrimination compared to pair discrimination (*p* = .02) and on intonation discrimination compared to stress discrimination (*p* = .002); there was a significant correlation between the total acoustic processing score and linguistic prosody score (*r* = .50, *p* = .03). The three patients with peripheral hearing deficits performed within the range of performance of patients without hearing deficits, suggesting that prosodic deficits were not attributable simply to peripheral hearing loss. None of the linguistic prosody subtest scores correlated with auditory short-term memory capacity, as indexed by digit span, although there was a correlation between pair discrimination and performance on the Trails B test in the PPA group as a whole (*r* = .36, *p* = .006).

On the emotional prosody test, the PNFA subgroup performed significantly worse than controls in total and on each of the individual emotions (Table 2). The LPA subgroup performed significantly worse than controls in total and on each of the individual emotions except surprise where there was a trend to worse performance. The small GRN-PPA subgroup did not perform significantly worse than controls on any of the emotions although there was a trend to worse performance on each of the emotions. There was no significant difference between the subgroups on any of the individual emotions. For the PPA cohort overall, sadness and surprise were best recognised and disgust and fear least well recognised; there were statistically significant differences in recognition performance for fear versus surprise (*p* = .03) and sadness (*p* = .02) and for disgust versus surprise (*p* = .046). The qualitative pattern of recognition performance for individual emotions was similar in patients and healthy controls (Table 2).

Performance on recognition of facial expressions was also impaired in the subgroup of 13 patients assessed on both modalities [mean (standard deviation) overall score 14.2 (3.4)/24; controls, 20.5 (1.9)/24]. However, patients’ performance on recognition of vocal emotions was significantly inferior (*p* = .02) to recognition of facial expressions, while control performance did not differ significantly between the two modalities. Furthermore, the pattern of patient performance for recognition of individual emotions varied between modalities: for facial expressions (in contrast to vocalisations), happiness was best recognised (mean 94% correct; chance 16%), followed by surprise (64%), anger, sadness, disgust (all 54%) and fear (37%).

### 3.2 Neuroimaging data

**Fig. 2:**
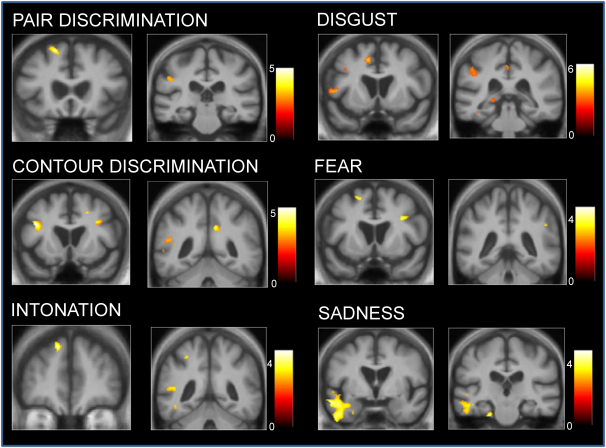
GM changes associated with performance on acoustic and linguistic prosody discrimination subtests (left panels) and emotional prosody recognition subtests (right panels) for the PPA cohort. Statistical parametric maps have been thresholded at *p* < .005 (uncorrected over the whole brain volume), inclusively masked by the region of disease-related atrophy (see text). Maps have been rendered on coronal sections from the study-specific average group T1-weighted MRI template image in DARTEL space. The colour bar adjacent to each panel indicates the range of t scores coded for that map. The left hemisphere is shown on the left side for all images.

As there was no overall difference in prosodic performance between the PPA subgroups, subgroups were combined in the VBM analysis. Anatomical data associated with performance on each of the prosody subtests for the combined PPA group are summarised in Table 3; statistical parametric maps of associated GM change are shown in Fig. 2. Whole-brain VBM analyses have been thresholded at *p* < .005 (uncorrected for multiple voxel-wise tests over the whole brain volume) with inclusive masking by the region of disease-related atrophy; clusters larger than 20 voxels are reported.

For the acoustic prosody subtests, pair discrimination score was positively associated with GM in left dorsal prefrontal, inferior parietal and posterior cingulate cortices; while contour discrimination score was positively associated with GM in bilateral inferior frontal and posterior temporal gyri, anterior and posterior cingulate cortex, and left inferior parietal cortex. For the linguistic prosody subtests, intonation discrimination score was positively associated with GM in left dorsal prefrontal cortex, posterior cingulate cortex, posterior superior temporal cortex and fusiform gyrus; no associations of stress discrimination performance were identified within the region of disease-related atrophy. For the emotional prosody subtests, GM associations were identified for recognition of the negative emotions disgust, fear and sadness: recognition of each of these emotions was positively associated with GM in left dorsal prefrontal cortex. In addition, disgust recognition was associated with GM in left inferior frontal cortex, anterior and posterior cingulate cortex, posterior, superior, inferior and mesial temporal cortices, left hippocampus, and right anterior insular and inferior parietal cortices; while fear recognition was associated with GM in right dorsolateral prefrontal and posterior superior temporal cortices and left visual association cortex, and sadness recognition was associated with GM in left orbitofrontal cortex, anterior superior, inferior and mesial temporal cortices and inferior parietal cortex.

## 4 Discussion

Here we have demonstrated impairments of receptive prosody in nonfluent PPA syndromes. Deficits were exhibited by all subgroups for acoustic, linguistic and affective dimensions of prosodic analysis. The finding of impairment at the level of the basic acoustic building blocks of prosodic contours and the correlation between acoustic and linguistic prosody performances argue for the involvement of early perceptual mechanisms that cascade to higher levels of prosodic processing in PPA. Whereas prosodic variation in syllables and words typically extends over tens to hundreds of milliseconds, prosodic contours typically extend over hundreds to thousands of milliseconds: the prosodic subtests used here (syllable pairs/word stress *vs* contour/intonation) might index the processing of prosodic structure over shorter versus longer timescales, respectively. Contour discrimination was significantly more impaired than pair discrimination and intonation discrimination was significantly more impaired than stress discrimination at the phrasal level: this pattern suggests that the representation of longer range prosodic structure may be relatively more vulnerable in PPA. While this pattern might be at least partly attributable to an associated working memory impairment, the lack of correlation between prosodic and short-term memory and executive performance on most of the tasks argues for an additional specific deficit of receptive prosody in PPA. Within the domain of affective prosody, recognition of certain emotions (in particular, disgust and fear) was relatively more impaired. Comparison of vocal emotion recognition with recognition of emotions in another modality (facial expressions) here suggested non-uniform involvement of emotion processing mechanisms between modalities in PPA: recognition of vocal emotions was significantly inferior to recognition of facial expressions in patients (but not healthy controls), and the relative degree of impairment of particular emotions differed for vocalisations versus facial expressions. Taken together, the data suggest a generic deficit of emotion recognition in PPA, but further suggest that this may be modulated by modality-specific (possibly perceptual) factors. Whereas vocal expressions of emotions such as sadness and surprise can be conveyed vocally from relatively coarse perceptual cues (e.g., large shifts in intensity or pitch), the perception of vocal expressions of other negative emotions is likely to be relatively more dependent on accurate encoding of fine-grained perceptual features (Juslin and Laukka, 2003; Hammerschmidt and Jürgens, 2007). Healthy subjects may be better able to exploit discriminatory acoustic features of emotional prosodic utterances, or alternatively, there may be an additional specific deficit in processing particular vocal emotions in PPA: the present data do not resolve this issue.

Perception of prosody has been little studied in degenerative disease. Impairments of emotional prosody processing have been documented in Huntington’s disease (Speedie et al., 1990; Snowden et al., 2008), Parkinson’s disease (Dara et al., 2008), Alzheimer’s disease (Taler et al., 2008) and frontotemporal dementia (right temporal lobe atrophy: Perry et al., 2001). The brain basis for prosodic deficits in these disorders remains largely unexplored. Studies of prosody in patients with stroke or functional magnetic resonance imaging (fMRI) studies in cognitively-normal individuals have implicated a predominantly right-sided (though often bilateral) distributed fronto-temporo-parietal network in the processing of emotional prosody, with less consistent lateralisation for the processing of linguistic prosody (e.g., Tong et al., 2005; Ethofer et al., 2006; Pell, 2006a, 2006b; Wildgruber et al., 2006; Beaucousin et al., 2007; Arciuli and Slowiaczek, 2007; Wiethoff et al., 2008; Ross and Monnot, 2008). The present findings in PPA corroborate this previous work, delineating a distributed network of areas associated with processing of different dimensions of linguistic and emotional prosody. While the findings here suggest predominantly left hemispheric associations, there is an important caveat in that the region of maximal disease involvement in the PPA syndromes is left lateralised: by restricting analysis to this leftward asymmetric disease region, we have delineated anatomical areas that are more likely to be true disease associations, but limited the potential to detect right hemispheric associations of prosodic processing. The cortical associations of acoustic and linguistic prosody processing identified here include areas (posterior temporal lobe, inferior parietal lobe) previously implicated in the perceptual analysis of nonverbal vocalisations, (Wildgruber et al., 2005, 2006; Gandour et al., 2007; Wiethoff et al., 2008; Ischebeck et al., 2008) and additional fronto-parietal circuitry that may be involved in attention, working memory and ‘mirror’ responses to heard vocalisations (Warren et al., 2005, 2006). Structures such as cingulate cortex that participate in generic attentional and related processes may be engaged particularly by demands for suprasegmental analysis of vocalisations (Knösche et al., 2005). Associations of emotional prosody processing were identified in a broadly overlapping network of frontal, temporal and parietal areas, including components of the limbic system. Within this network, certain areas may have relative specificity for recognition of particular negative emotions. The insula and mesial temporal structures are involved in recognition of emotions (in particular, disgust) in various modalities (Phillips et al., 1997; Hennenlotter et al., 2004; Jabbi et al., 2008). Anterior temporal cortical areas have been previously implicated in visual processing of negative emotions (in particular, sadness) in both healthy subjects (Britton et al., 2006) and patients with dementia (Rosen et al., 2006); these areas are likely to have a role in decoding social signals. Performance on several of the prosodic subtests here was associated with GM changes in ‘visual’ cortical areas: this apparently paradoxical finding may reflect cross-modal influences (e.g., visual imagery) on the processing of prosodic signals (Brosch et al., 2009; Foxton et al., 2010). Taken together, the present neuroanatomical findings are consistent with an emerging hierarchical and multidimensional organisation of prosodic processing (Wildgruber et al., 2006).

Whereas deficits of speech processing have been emphasised on clinical and neuroanatomical grounds in PPA, this study suggests a more general defect (or defects) of vocal signal processing. Speech prosody serves a key ‘metalinguistic’ function in human communication, and deficits of prosody processing therefore have potentially important clinical consequences. Indeed, as PPA typically affects the left hemisphere initially, receptive dysprosodia may become more clinically significant with increasing right hemisphere involvement as the disease evolves. In future work, it will be essential to address prosody processing in the third canonical variant of PPA, SD, in order to arrive at a complete understanding of this important class of nonverbal vocal signals in the language-based dementias. In addition, the experimental battery used here was designed to provide an initial overall assessment of receptive prosody, sampling in each of the key prosodic dimensions (acoustic, linguistic and affective): analysis of specific components of each of these dimensions will be required in order to understand the mechanisms of prosodic dysfunction in PPA syndromes. Further longitudinal studies with larger PPA cohorts are needed to establish the natural history of prosody impairment in PPA in relation to linguistic deficits, to define prosodic signatures of particular PPA subgroups, to explore related aspects of complex sound processing across the PPA spectrum and to define the brain basis of prosodic deficits in detail.

## Figures and Tables

**Table 1:** Demographic and baseline neuropsychological data.

	ALL PPA	PNFA	LPA	GRN-PPA	Controls
Number of subjects	19	11	5	3	14
Age (years)	68.6 (7.9)	72.8 (6.5)	63.1 (4.4)	62.0 (8.5)	68.2 (4.8)
Gender (M:F)	12:7	7:4	3:2	2:1	7:7
Duration (years)	4.9 (1.6)	5.3 (1.9)	4.5 (1.0)	4.3 (.6)	N/A
Warrington synonyms test (/50)	36.2 (1.5)^a^	39.6 (1.8)^a^	31.4 (2.4)a,b	31.7 (6.7)^a^	48.0 (.3)
Trail making test A (scaled score)	3.8 (.6)^a^	2.9 (.4)^a^	5.7 (4.0)^a^	4.2 (2.7)^a^	10.1 (.5)
Trail making test B (scaled score)	3.0 (.5)^a^	3.1 (.5)^a^	2.0 (.8)^a^	4.5 (4.8)^a^	10.7 (.5)
Digit span forwards	4.1 (.3)^a^	4.4 (.4)^a^	4.6 (1.5)^a^	2.0 (1.0)a,c,d	6.9 (.1)

Mean (standard deviation) values shown.

a *p* < .05 disease group worse than controls.

b *p* < .05 LPA worse than PNFA.

c *p* < .05 GRN-PPA worse than PNFA.

d *p* < .05 GRN-PPA worse than LPA.

**Table 2:** Acoustic processing, linguistic prosody and emotional prosody data.

	All PPA	PNFA	LPA	GRN-PPA	Controls
Acoustic processing					
Pair discrimination (/12)	9.3 (1.6)^a^	9.5 (1.8)^a^	8.2 (1.1)a,b,c	10.0 (1.0)^a^	11.4 (.7)
Contour discrimination (/12)	7.8 (2.5)^a^	7.5 (2.9)^a^	7.8 (2.6)^a^	9.0 (.0)^a^	11.5 (.5)

Total (/24)*	17.1 (3.4)^a^	17.0 (3.8)^a^	16.0 (3.5)^a^	19.0 (1.0)^a^	22.9 (1.0)

Linguistic prosody					
Stress discrimination (/14)	12.1 (2.6)^a^	12.5 (1.8)	10.2 (3.8)a,c	14.0 (.0)	13.9 (.5)
Intonation discrimination (/14)	9.1 (2.5)^a^	9.6 (2.9)^a^	8.0 (2.2)a,b	9.0 (1.0)^a^	13.4 (1.0)

Total (/28)**	21.2 (4.0)^a^	22.1 (4.0)^a^	18.2 (3.8)^a^	23.0 (1.0)^a^	27.2 (1.4)

Emotional prosody					
Sadness (%)	65.8 (32.5)^a^	75.0 (29.6)^a^	55.0 (27.4)^a^	50.0 (50.0)	98.2 (6.7)
Surprise (%)	60.5 (29.2)^a^	61.4 (30.3)^a^	55.0 (37.1)	66.7 (14.4)	91.1 (15.8)
Anger (%)	46.1 (35.6)^a^	50.0 (40.3)^a^	40.0 (28.5)^a^	41.7 (38.2)	85.7 (16.2)
Happiness (%)	44.7 (24.4)^a^	40.9 (23.1)^a^	45.0 (32.6)^a^	58.3 (14.4)	80.4 (20.0)
Disgust (%)	31.6 (23.3)^a^	38.6 (20.5)^a^	15.0 (13.7)^a^	33.3 (38.2)	64.3 (25.4)
Fear (%)	30.3 (27.1)^a^	31.8 (22.6)^a^	20.0 (32.6)^a^	41.7 (38.2)	78.6 (29.2)

Total (/24)***	11.1 (3.7)^a^	11.8 (2.9)^a^	9.2 (2.8)^a^	11.7 (7.1)	19.9 (2.6)

Mean (standard deviation) values shown. *chance score = 12; **chance score = 14; ***chance score = 4.

a *p* < .05 disease group worse than controls.

b *p* < .05 LPA worse than PNFA.

c *p* < .05 LPA worse than GRN-PPA.

**Table 3:** Summary of anatomical regions associated with prosodic performance across the PPA group.

Prosody subtest	Local maximum [x y z] (mm)^a^	Z score	Region	Brodmann area
Acoustic				
Pair discrimination	−15 18 46	3.76	Dorsal PFC	BA9
−15 −54 3	3.41	PCC	BA31
−33 −60 30	2.93	IPL	BA39
−46 −28 28	2.80	BA40
Contour discrimination	−38 11 24	3.95	IFG	BA44
−28 −69 21	3.59	IPL	BA39
9 −48 30	3.44	PCC^b^	BA31
30 9 25	3.38	IFG^b^	BA44
12 −13 33	3.19	ACC^b^	BA24
−40 −55 4	2.99	Post MTG	BA21
52 −34 −8	2.94	Post MTG^b^	BA21

Linguistic				
Intonation	−2 −34 37	3.67	PCC	BA31
−12 42 36	3.57	Dorsal PFC	BA9
−46 −46 12	3.09	Post STG/STS	BA22
−40 −48 −8	3.05	Fusiform gyrus	BA37

Emotional				
Disgust	−42 −10 12	4.26	Frontal operculum	BA43
−8 11 42	3.69	SMA	BA6
−32 −24 −5	3.58	Hippocampus	–
−36 0 31	3.42	Premotor	BA6
0 −36 37	3.41	PCC	BA31
−8 −4 40	3.32	ACC	BA24
38 16 10	3.26	Ant insula^b^	–
−33 30 −6	3.24	OFC	BA11
−52 −43 10	3.23	Post STG/STS	BA22
−20 −40 −9	3.23	PHG	BA36
56 −52 21	3.17	IPL^b^	BA39
−40 −40 −23	3.06	Fusiform gyrus	BA37
Fear	54 −46 18	3.58	Post STG/STS^b^	BA22
−28 −81 0	3.52	Visual association cortex	BA19
−16 8 49	3.08	Premotor	BA8
32 8 27	3.07	Dorsal PFC^b^	BA46
Sadness	−20 −21 −27	3.58	PHG	BA36
−34 −60 37	3.50	IPL	BA39
−52 −4 −18	3.47	Ant STG/STS	BA38/22
−3 41 45	3.23	Dorsal PFC	BA9
−38 20 −21	3.20	OFC	BA11
−45 −45 −11	2.88	ITG	BA20/37

Data have been thresholded at *p* < .005 uncorrected and masked by the region of disease-related atrophy for the PPA group as a whole versus healthy controls; all clusters >20 voxels in size are reported.

Key: ant, anterior; ACC, anterior cingulate cortex; IFG, inferior frontal gyrus; IPL, inferior parietal lobe; ITG, inferior temporal gyrus; MTG, middle temporal gyrus; OFC, orbitofrontal cortex; PCC, posterior cingulate cortex; PFC, prefrontal cortex; post, posterior; PHG, parahippocampal gyrus; SMA, supplementary motor area; STG, superior temporal gyrus; STS, superior temporal sulcus.

a Coordinates are in DARTEL space.

b Indicates R hemisphere (all others within L hemisphere).
